# Right single lung transplantation using an inverted left donor lung: interposition of pericardial conduit for pulmonary venous anastomosis - a case report

**DOI:** 10.1186/s12890-020-1075-4

**Published:** 2020-02-19

**Authors:** Haruchika Yamamoto, Kentaroh Miyoshi, Shinji Otani, Takeshi Kurosaki, Seiichiro Sugimoto, Masaomi Yamane, Shinichi Toyooka, Motomu Kobayashi, Takahiro Oto

**Affiliations:** 1 Thoracic Surgery, Okayama University Hospital, Okayama, Japan; 2 Organ Transplant Center, Okayama University Hospital, Okayama, Japan; 3 Anesthesiology, Okayama University Hospital, Okayama, Japan

**Keywords:** Inverted lung transplantation, Pericardial conduit, Pulmonary venous anastomosis, Vessel formation

## Abstract

**Background:**

Lung transplantation (LTx) is still limited by the shortage of suitable donor lungs. Developing flexible surgical procedures can help to increase the chances of LTx by unfolding recipient-to-donor matching options based on the pre-existing organ allocation concept. We report a case in which a successful left-to-right inverted LTx was completed using the interposition of a pericardial conduit for pulmonary venous anastomosis.

**Case presentation:**

A left lung graft was offered to a 59-year-old male who had idiopathic pulmonary fibrosis with predominant damage in the right lung. He had been prescribed bed rest with constant oxygen inhalation through an oxymizer pendant and had been on the waiting list for 20 months. Considering the condition of the patient (LAS 34.3) and the scarcity of domestic organ offers, the patient was highly likely to be incapable of tolerating any additional waiting time for another donor organ if he was unable to accept the presently reported offer of a left lung. Eventually, we decided to transplant the left donor lung into the right thorax of the recipient. Because of the anterior-posterior position gap of the hilar structures, the cuff lengths of the pulmonary veins had to be adjusted. The patient did not develop any anastomotic complications after the transplantation.

**Conclusions:**

A left-to-right inverted LTx is technically feasible using an autologous pericardial conduit for pulmonary venous anastomosis in selected cases. This technique provides the potential benefit of resolving challenging situations in which surgeons must deal with a patient’s urgency and the logistical limitations of organ allocation.

## Background

The development of flexible surgical procedures has increased the chances of lung transplantation (LTx) by unfolding recipient-to-donor matching options based on the pre-existing organ allocation concept; surgery to liberate the grafting side is one such example. In particular, a single inverted left-to-right LTx may be considered under the following conditions: 1) despite a right-side-predominant lung dysfunction in the recipient candidate, the available donor organ options are limited to the use of a left single donor lung, based on evidence of right donor lung injury or the need for the donor lung to be shared with another candidate with waitlist priority; or 2) a left side transplantation in the recipient is impossible because of a past history of thoracic surgery [1]. The need to adjust the positional relationships because of the take-off angle mismatch of the hilar structures is one of the key challenges in inverted transplantation. Herein, we report a case of successful inverted left-to-right LTx with pulmonary venous anastomosis using pericardial conduit interposition for the venous anastomosis.

## Case presentation

**Fig. 1:**
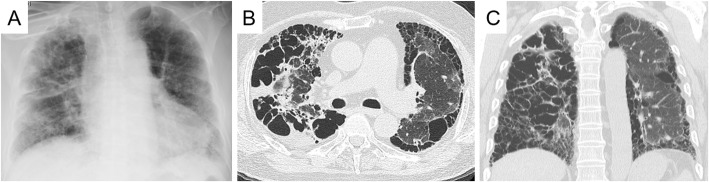
**a**) Preoperative chest X-ray and **b**), **c**) chest computed tomographic images of the recipient. The preoperative chest X-ray and chest computed tomographic images revealed heterogeneous disease progression, with predominant damage in the right lung

A 59-year-old male patient with idiopathic interstitial fibrosis who had been receiving long-term oxygen therapy was considered for a single LTx. A chest computed tomography (CT) examination and ventilation-perfusion scintigraphy (ventilation: right/left = 55%/45%; perfusion: right/left = 35%/65%) revealed heterogeneous disease progression, with predominant damage in the right lung (Fig. 1). The results of an arterial blood gas test and a 6-min walk test were a pO2 of 106.5 mmHg, a pCO2 of 55 mmHg, and 156 m, respectively, under 7 L/min of oxygen inhalation using an oxymizer pendant. The patient’s activity level was restricted to moving from bed to a wheelchair while he continued to receive rehabilitation, and he had been on the waitlist for 20 months.

**Fig. 2:**
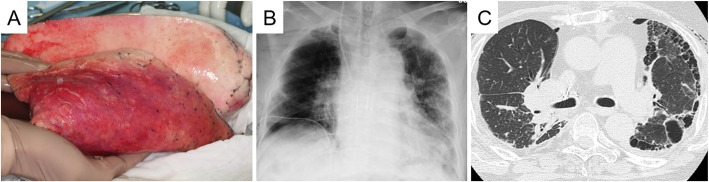
**a** Photograph of the left graft. Broad edematous lesions became apparent in the left lower lobe of the graft lung after flushing. **b** Chest X-ray and **c** chest computed tomographic image on postoperative day 7. The chest X-ray and chest computed tomographic images obtained on postoperative day 7 revealed the prompt amelioration of the edematous changes in the donor lower lobe in the reverse position and the adaptation of the left donor lung in the recipient right chest cavity without the development of atelectasis

A 50-year-old female donor died of hypoxic brain damage. A plain chest x-ray revealed no marked infiltration in either lung field. An arterial blood gas analysis revealed a PaO2/FiO2 ratio of 510, although inspection after lung flushing during the procurement surgery revealed broad edematous changes and a poor compliance of the left lower lobe (Fig. 2a). A right-sided LTx, rather than a left-sided LTx, was the favorable option, considering the heterogeneity of our patient’s lung injury. However, he had to share the offered lungs with another candidate who was prioritized on the waitlist and also needed a right LTx. Because of the seriousness of his condition and the scarcity of organ donors in Japan [2], we considered that the patient might not be capable of tolerating any additional waiting time if the presently reported offer of the left lung could not be accepted. The recipient’s CT images and lung perfusion function tests showed that the deterioration in the right lung was more significant than that in the left lung; in addition, the graft was not a perfect lung but a marginal lung. Therefore, we chose to leave the left lung with better function and to transplant the graft into the right thoracic cavity so as to maximize the achievable total lung function. As for the pulmonary edema in the left lower lobe of the donor lung, we anticipated early re-conditioning because of the reversed positioning in the recipient thorax. Taking all aspects into consideration, we eventually decided to transplant the offered left donor lung into the right thorax of the recipient.

**Fig. 3:**
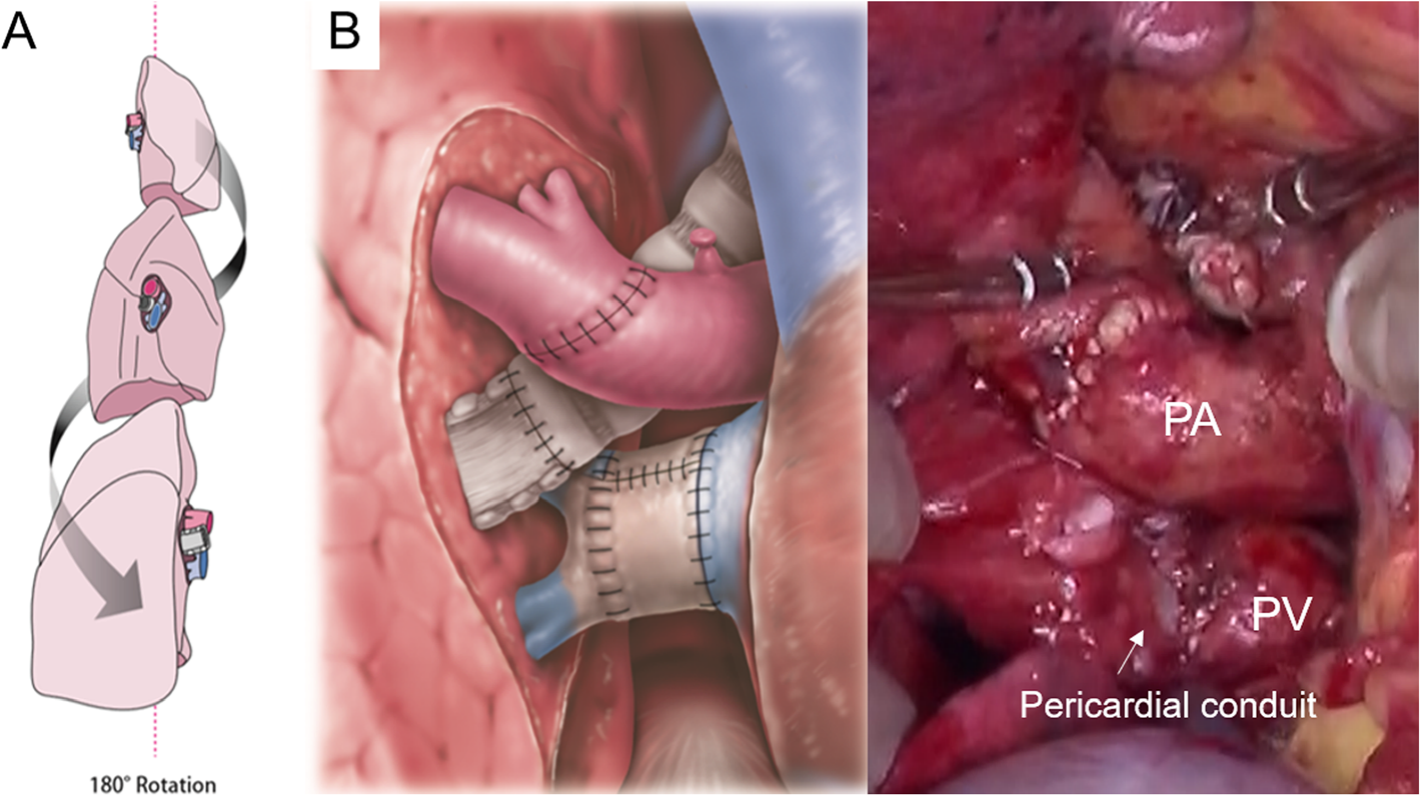
**a** Schema of the inverted left lung. **b** Schema and photograph of the anastomosis. Because of the anterior-posterior position gap between the donor and recipient pulmonary veins, a venous cuff extension using autologous pericardial conduits was necessary. However, no conduit was required for the pulmonary artery anastomosis, as both the recipient and donor cuffs were sufficiently long. Bronchial anastomosis was performed with membranous-to-cartilaginous apposition

The operation was performed under cardiopulmonary bypass (CPB) through a right semi-clamshell incision. The circulatory dynamics were unstable during the left PA clamp test. CPB was used to obtain cardiac drainage, and a surgical field in which the central hilar region was easily accessible was created. The interatrial groove was dissected as much as possible to facilitate the clamping of the pulmonary veins as proximally as possible. The recipient pulmonary artery was transected at the distal level of the first branch. A maximal length of the donor pulmonary artery and atrial cuff were secured. The donor left lung was placed in the recipient right thorax with a 180° rotation around its superior-inferior axis from its anatomic position (Fig. 3a). The anastomoses were performed from the dorsal side in the order of the pulmonary vein, bronchus, and pulmonary artery. Because of the anterior-posterior position gap between the donor and recipient pulmonary veins, a venous cuff extension was necessary so as to secure a cuff length sufficient for anastomosis. The veins were separately anastomosed using autologous pericardial conduits on the anterior and posterior sides. The anterior wall and posterior wall were built with the pedicle pericardium and free pericardium from the donor, respectively, using a running 6–0 polypropylene suture. End-to-end bronchial anastomosis was performed, with membranous-to-cartilaginous apposition. The donor pulmonary artery was located superiorly and anteriorly, while the recipient’s was located inferiorly and posteriorly. To resolve this problem, it was necessary to dissect the recipient pulmonary artery diagonally and to translocate it to an anterior position. However, no conduit was required for the pulmonary artery anastomosis, since both the recipient and the donor cuffs were sufficiently long. After completing the anastomosis (Fig. 3b), reperfusion was successfully achieved without complications. Neither kinking of the pulmonary vessels nor congestion of the transplanted lung were observed. The CPB time, operative time and ischemic time were 231 min, 421 min and 514 min, respectively. The blood loss was 1730 mL; 0 mL of red blood cells, 1440 mL of frozen fresh plasma, and 400 mL of platelet concentrates were administered during the operation. No postoperative anticoagulation was required.

The patient developed grade 3 primary graft dysfunction but recovered quickly. A chest CT examination on postoperative day 7 revealed prompt amelioration of the edematous changes in the donor lower lobe in the reverse position and adaptation of the left donor lung in the recipient right chest cavity without the development of atelectasis (Fig. 2b). The patient was hemodynamically stable and was weaned from the mechanical ventilator 4 days after the surgery. He was diagnosed as having antibody-mediated rejection (AMR) at 89 and 158 days after LTx, necessitating several courses of intensive immunosuppressive treatment and prolonging the period of hospitalization, but the patient was eventually discharged from the hospital 6 months after the LTx. Unfortunately, the repeated AMR episodes resulted in reluctant *Aspergillus* pneumonia, and the patient died 260 days after surgery. However, no radiological or physiological evidence of anastomosis problems was seen throughout the postoperative course.

## Discussion and conclusions

In the present case, we used an organ with limited availability successfully and effectively by explanting the recipient’s less-functional right lung and transplanting a donor lung from the opposite side (left lung). The position gap of the hilar structures was overcome using a pericardial conduit for pulmonary venous anastomosis. No anastomotic complications were encountered, and the morphological discrepancy between the inverted left lung graft and the recipient’s right chest cavity posed no problems post-transplantation.

In inverted lung transplantation [1], the graft bronchus is located anteriorly and the graft pulmonary artery and vein are located posteriorly to the recipient’s hilar structures. The rigidity of the bronchus usually determines the positional relationship between the hilar structures of the graft and recipient. Thus, the cuff lengths of the remaining pulmonary artery and veins must be adjusted. A long pulmonary arterial cuff is easily secured by leaving the recipient’s main pulmonary artery as distally as possible. However, the same is not the case for the pulmonary venous cuff in a single LTx in which the heart and opposite lung are also utilized for another patient.

We resolved the issue by interposing a conduit constructed with the donor’s pericardium. The conduit attachment procedure was performed on a back table, where it was easy to handle the graft and to manage the procedure. Autologous pericardial conduit interpositioning has been reported as an effective technique for cuff extension in both cadaveric and living donor lobar lung transplantation [3–5].

This technique has several potential limitations. There is some risk of kinking of the vascular anastomosis, of complications related to the bronchial membranous-to-cartilaginous anastomosis, and of atelectasis due to an imperfect fit of the donor lung in the recipient’s chest cavity.

On the other hand, as a potential secondary benefit, the edematous portion of the lower lobe of the grafted lung was inverted to the anterior side, enabling reconditioning from the edema to be obtained at relatively early during the postoperative period.

In conclusion, a left-to-right inverted transplantation is technically feasible in selected cases using an autologous pericardial conduit for pulmonary venous anastomosis. This technique provides the potential benefit of resolving challenging situations in which surgeons must to deal with a patient’s urgency and the logistical limitations of organ allocation.

## Data Availability

Not applicable.

## Abbreviations

AMR: Antibody-mediated rejection
CPB: Cardiopulmonary bypass
CT: Computed tomography
LTx: Lung transplantation
